# Burnout of Physicians Working in Primary Health Care Centers under Ministry of Health Jeddah, Saudi Arabia

**DOI:** 10.7759/cureus.1877

**Published:** 2017-11-25

**Authors:** Khalid Bawakid, Ola Abdulrashid, Najlaa Mandoura, Hassan Bin Usman Shah, Adel Ibrahim, Noura Mohammad Akkad, Fauad Mufti

**Affiliations:** 1 Deputy Director of General Directorate of Health Affairs for Public Health Division, Jeddah, Directorate of Health Affairs for Public Health Division, Jeddah; 2 Head of Research Unit, Directorate of Health Affairs for Public Health Division, Jeddah; 3 Research Department, Directorate of Health Affairs for Public Health Division, Jeddah; 4 Research Department, Directorate of Health Affairs, Public Health Division, Jeddah; 5 Primary Health Care Centre, PHCC Gholail, Jeddah

**Keywords:** abbreviated maslach burnout inventory, burnout, emotional exhaustion, physicians, stress

## Abstract

Introduction

The levels of physicians' job satisfaction and burnout directly affect their professionalism, punctuality, absenteeism, and ultimately, patients' care. Despite its crucial importance, little is known about professional burnout of the physicians in Saudi Arabia. The objectives of this research are two-fold: (1) To assess the prevalence of burnout in physicians working in primary health care centers under Ministry of Health; and (2) to find the modifiable factors which can decrease the burnout ratio.

Methodology

Through a cross-sectional study design, a representative sample of the physicians working in primary health care centers (PHCCs) Jeddah (n=246) was randomly selected. The overall burnout level was assessed using the validated abbreviated Maslach burnout inventory (aMBI) questionnaire. It measures the overall burnout prevalence based on three main domains i.e., emotional exhaustion, depersonalization, and personal accomplishment. Independent sample T-test, analysis of variance (ANOVA), and multivariate regression analysis were performed using Statistical Package for the Social Sciences (SPSS Version 22, IBM, Armonk, NY).

Results

Overall, moderate to high burnout was prevalent in 25.2% of the physicians. Emotional exhaustion was noted in 69.5%. Multivariate regression analysis showed that patient pressure/violence (p <0.001), unorganized patients flow to clinics (p=0.021), more paperwork (p<0.001), and less co-operative colleague doctors (p=0.045) were the significant predictors for high emotional exhaustion. A positive correlation was noted between the number of patients per day and burnout. The patient’s pressure/violence was the only significant independent predictor of overall burnout.

Conclusion

Emotional exhaustion is the most prominent feature of overall burnout in the physicians of primary health care centers. The main reasons include patient’s pressure/violence, unorganized patient flow, less cooperative colleague doctors, fewer support services at the PHCCs, more paperwork, and less cooperative colleagues. Addressing these issues could lead to a decrease in physician’s burnout.

## Introduction

Occupational burnout is a relatively new concept introduced in the 1970s. It covers a wide range of manifestations, ranging from exhaustion, frustration, anger, and negative attitudes towards work and patients. This includes ineffectiveness, absenteeism, and even wanting to leave leaving the job [1-2].

Generally, burnout is seen in the occupations demanding interaction with people; hence, it is common in health care sector [3-5]. Stress and burnout are substantial problems for health professionals. Although rewarding, caring for patients is demanding and stressful. Physicians have long working hours and are frequently exposed to deaths and sufferings. The above-mentioned reasons contribute to a physician’s burnout [6-8].

Physician burnout is a growing epidemic with estimates showing every third physician to experience at least one dimension of burnout [7, 9-10]. Studies prove that work that once seemed important, meaningful, and challenging to the physicians becomes unpleasant and meaningless [10-11]. Maslach, et al. [11] describes burnout as positive energy turning into exhaustion, involvement/commitment into cynicism, and efficacy into ineffectiveness.

Burnout is commonly conceptualized as a multidimensional syndrome consisting of three components [11-12]: emotional exhaustion (EE), where emotional resources of the workers are depleted, and they feel that they are no longer able to give their best at a psychological level; depersonalization (DP), where workers develop negative cynical attitude and feelings about one’s clients; and reduced personal accomplishment (PA), a tendency to negatively evaluate oneself [11, 13].

Meta-analysis shows burnout to be common among front-line physicians [12-13]. Primary health care physicians (PHCCs) represent the first line of providing health care services in any country [5, 14]; they spend a considerable part of their time dealing with emergencies/difficult cases, and they have to face the aggressiveness of the attendants [7, 12]. Other stressful factors include extensive workload, shortage of staff and resources, job insecurity, lack of incentives, rapidly changing clinical environment and circumstances, unsafe working environment, and conflicts with the patients and colleagues [3, 5, 14]. Any negligence or medical error by the health professional can be detrimental to the patient, which doubles their stress [15]. Moreover, physician’s tendency to give suboptimal attention to self-wellness increases the chances of burnout [4, 8, 14]. These stressors can negatively affect their performance, which ultimately leads to exhaustion and burnout [14-15].

Globally, the healthcare organizations have realized the need to address physician’s burnout as it not only affects the suffering doctors but also reflects the quality of care provided by these doctors [13-15]. It is therefore important to understand and identify the factors that contribute to the increase in burnout in physicians as well as to try to minimize them. Not much research has been done on the professional burnout among the front-line physicians working in Jeddah. Therefore, the current study aims at estimating the prevalence of job burnout, and to identify its determinants among physicians working in the PHCCs of Ministry of Health (MoH), Jeddah.

## Materials and methods

A cross-sectional, interview-based survey was conducted in primary health care centers in Jeddah, working under Ministry of Health. The duration of the study was of four months from Oct 1, 2016, to Jan 31, 2017. The sample size was calculated using the online EpiTools sample size calculator. Based on the results of other regional studies [10], the mean burnout score calculated was 11 with a standard deviation of four. Using these figures, we kept the level of significance at 95% and desired precision at 0.5; the calculated sample size was found to be 246. We approached 266 physicians, but 20 doctors did not give consent; a response rate of around 92% was noted. Physicians included general practitioners (GPs), family medicine specialists (FPs), and dentists. FPs are specialist/consultant doctors who have finished and cleared exit exam after four years of post-graduate residency training in family medicine, while general practitioners are simple under-graduate doctors with no post-graduate training in family medicine. We selected the physicians proportionally using stratified random sampling technique.

Jeddah PHCCs are divided into five geographical regions. The region having the maximum number of physicians was given maximum proportion in the sampling. All the physicians present in that facility on the day of data collection were interviewed. A widely used, reliable, and validated instrument (considered to be ‘Gold standard’ in this field) was used to gather the data in this survey to measure the overall burnout i.e., abbreviated Maslach burnout inventory (aMBI) [11, 16]. The validity of the aMBI is well established [16], and its internal consistency of the dimensions, using Cronbach’s alpha, are: emotional exhaustion = 0.74; depersonalization = 0.69; personal accomplishment = 0.71. Response options for the items were between zero (never) and six (every day). Responses were added to form a score for each subscale, thus giving each participant three scores for the three components of burnout as given below in Table 1:

**Table 1:** Scores for three components of burnout.

Burnout components	Scores range	Interpretation
Emotional exhaustion	Total (0-18)	Higher scores indicate greater emotional exhaustion and greater burnout
Depersonalization	Total (0-18)	Higher scores indicate greater depersonalization and greater burnout
Personal Accomplishment	Total (0-18)	Higher scores indicate greater personal accomplishment and less burnout

Using the recommended guidelines, overall burnout was determined by adding scores of emotional exhaustion and depersonalization (>75% percentiles) [17-18].

Data analysis was done using Statistical Package for the Social Sciences (SPSS Version 22, IBM, Armonk, NY). Bivariate analysis was performed using independent sample T-test and analysis of variance (ANOVA). Multivariate regression was performed to determine significant predictors of burnout in the physicians. P-value <0.05 was considered as an indication of significance.

Physicians who were employees of MoH working in Primary Health Care centers for more than six months and willing to participate were selected for this study. Ethical approval was taken from the ethical committee of the Directorate of Health Affairs, Jeddah and Ministry of Health (H-02-J-002-00762). Before the interview, verbal consent was taken from the physicians and confidentiality of data was ensured.

## Results

The study population consisted of 246 physicians working in the primary health care centers. Mean age of the physicians was 35 ± 7.6 years. The average weekly working days were 5.5 ± 1.0 with 8.8 ± 1.1 hours per day. The average daily patients in outpatient department (OPD) for each physician were around 30. The majority of physicians were Saudi general practitioners and family medicine specialist doctors (Table 2). Other demographic data have been provided in Table 2.

**Table 2:** Descriptive statistics.

Variable		n (Percentage)
Gender	Male	105 (42.6%)
	Female	141 (57.3%)
Specialty	Family Medicine	52 (21.1%)
	General Practitioner	164 (66.7%)
	Dentists/ Others	30 (12.2%)
Nationality	Saudi	228 (92.6%)
	Non-Saudi	18 (7.3%)
Marital status	Single	49 (19.9%)
	Married	190 (77.2%)
	Divorced	6 (2.4%)
	Widow	1 (0.4%)
Children	No child	74 (30.1%)
	< 3	112 (45.5%)
	> 3	60 (24.4%)

The physicians were not authorized by MoH to perform their duties outside PHCC (n=54, 22.2%) or in evening shifts (n=22, 8.9%). Most of the PHCC physicians were not satisfied with the restroom (82.1%) and paperwork (81.4%) in the center. Also, the majority of the physicians (73.9%) thought that the PHCC’s administrative department was slow. The mean scores with standard deviations (SD) of overall burnout and subscales are given in Table 3.

**Table 3:** Scores of abbreviated Maslach burnout inventory (aMBI) and its sub-scales.

Scale/ Subscale	Means (SD)	Min- Max	Percentiles		
			25^th^	50^th^	75^th^
Emotional exhaustion + depersonalization	17.28 (±8.71)	0- 36	11.00	17.00	25.00
Emotional exhaustion	11.60 (±4.70)	0-18	8.00	13.00	15.00
Depersonalization	5.66 (±5.20)	0-18	1.00	5.00	10.00
Reduced personal accomplishment	14.44 (±3.66)	0-18	13.00	15.00	17.00

According to the calculated 75th percentile, the overall prevalence of burnout among the physicians working in PHCCs was 25.2% (Table 4). Moderate to severe emotional exhaustion was found to be in 69.5% of the physicians. However, no significant difference (p=0.375) was noted between the overall burnout status of GPs (no or low burnout found in 76.8%) and FPs (no or low burnout found in 67.3%).

**Table 4:** Burnout status.

Burnout	Category of burnout	n (%)
Overall burnout	No or low burnout	184 (74.8)
	Moderate to High burnout	62 (25.2)
Emotional exhaustion	No or mild exhaustion	75 (30.4%)
	Moderate to severe	171 (69.5%)
Depersonalization	No or mild exhaustion	182 (74.0%)
	Moderate to severe	64 (26.0%)
Reduced personal accomplishment	Low burnout	216 (87.8%)
	Moderate to severe burnout	30 (12.2%)

**Figure 1:**
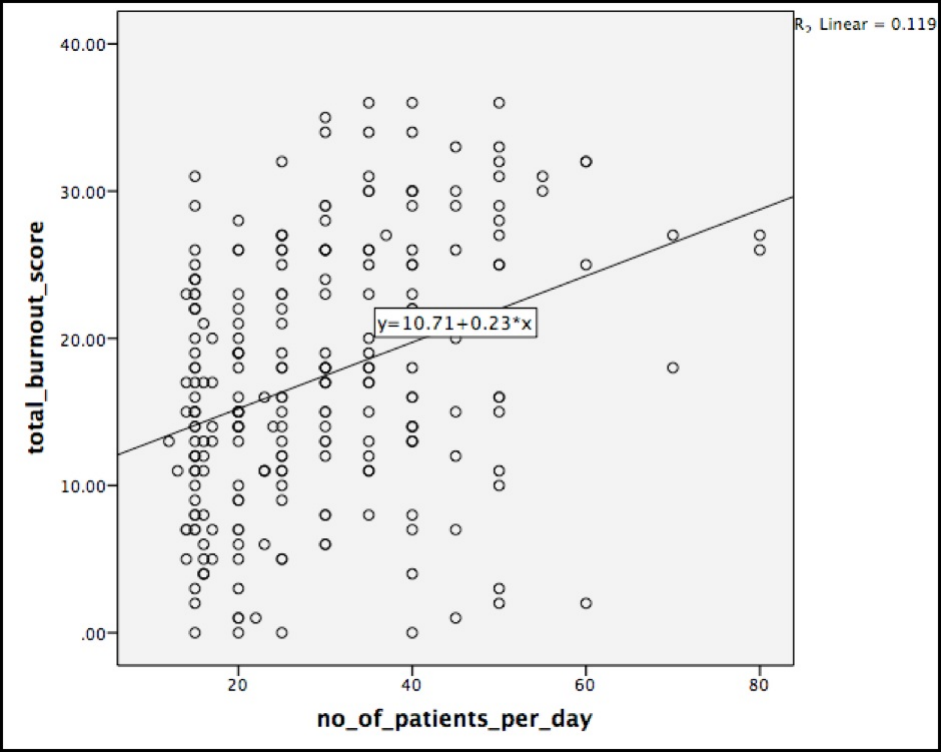
Daily patients in outpatient department (OPD) and total burnout score

Figure 1 shows a number of patients per day to be a positive predictor of overall burnout. (p < 0.001). Daily patients in OPD explains around 12% of the variance in overall burnout. 

The first step of multivariate regression analysis included using hierarchical regression and controlling for demographic factors (e.g., gender, specialty, marital status). The number of working days, hours per day, and number of patients per day were included in the second step of the analysis. It concluded that the significant predictors for high emotional exhaustion were patient pressure/violence (p <0.001), unorganized patient flow to clinics (p=0.021), more paperwork (p<0.001), and less cooperative colleagues/doctors (p=0.045) (Table 5).

**Table 5:** Multivariate regression model for emotional exhaustion

Variable	Standardized coefficient (B)	T-test	P value	95% Confidence Interval	
				Lower bound	Upper bound
Constant	5.246		<0.001	3.640	11.582
Patient pressure/violence	0.396	7.063	<0.001	1.774	3.147
Patient flow organization	-0.131	-2.330	0.021	-1.342	-0.112
PHCC managers' cooperation	0.113	1.907	0.058	-0.021	1.298
Paperwork	0.267	4.743	<0.001	0.679	1.644
Colleague doctors' cooperation	0.115	2.017	0.045	0.016	1.386

After bivariate analysis using T-test and ANOVA, only the statistically significant variables were included in multivariate analysis. Multivariate linear regression (Table 6) was performed to determine the factors responsible for burnout in the physicians.

**Table 6:** Multivariate regression model for overall burnout.

Variable	Standardized coefficient (B)	T-test	P-value	95% Confidence Interval	
				Lower bound	Upper bound
Constant	7.611		<0.001	3.640	11.582
Patient pressure/violence	0.368	6.367	<0.001	2.932	5.558
Easy to consult with colleagues	-0.109	-1.904	0.058	-2.955	0.050
PHCC managers' cooperation	0.100	1.694	0.092	-0.171	2.270
Paper work	0.124	1.903	0.058	-0.035	2.041
Administrative transactions	0.117	1.791	0.075	-0.099	2.082

As shown in Table 6, only one factor had a significant correlation with overall burnout, i.e., patient pressure, which is an independent risk factor for burnout. No statistically significant correlation was noted between burnout and consultation with the colleagues, cooperative PHCC managers, amount of paperwork done in PHCC, and slow administrative transactions. 

## Discussion

Burnout is considered an epidemic in the healthcare sector as healthcare professionals work in emotionally demanding situations. They are exposed to their clients’ psychological, socioeconomic, and physical problems resulting in the development of burnout [14-15]. The findings of this study gave insights into identifying the specific stressors and reasons of burnout in the physicians working in PHCCs, which are not only affecting their health but also compromising the quality of medical care delivered [15, 17].

The physicians working in PHCCs have to face a constant stress leading to burnout because of different reasons, depending on the organizational and personal context [10, 18-19]. High level of burnout in physicians is reported in many studies; ranging from 19% to 47% compared to 18% for the general employed population [20]. The prevalence of burnout reported in the physicians working in developed countries like Switzerland, Italy and France ranged from 30 to 50%, mostly because of high patient expectations/demands and fear of being sued [21]. A study conducted in Malaysia shows burnout prevalence to be around 27% [17]. A study conducted in Egypt by Yousef, et al. among resident physicians showed that 63.1% of the responding residents met the criteria for burnout; high burnout was because of the increased workload and frequent duties [22]. Our study results show an overall burnout rate of 25.2% which is much less than some developed countries. However, these burnout percentages were higher than the ones among physicians working in Yemen (11.7%), Qatar (12.6%) [23], and China (12%). This decrease in the burnout was mainly due to less patient load, less aggressive patients, and better healthcare facilities [24].

A study conducted in Japan showed high emotional exhaustion in 22% physicians and 11% with high level of depersonalization; 62% had a low level of personal accomplishment [25]. Similarly, a study conducted on family physicians of 12 European countries showed emotional exhaustion to be around 43%, 35% for depersonalization, and 32% for low personal accomplishment [26-27]. However, these figures did not match our study findings where the majority of physicians reported to be emotionally exhausted (70%), depersonalization in 26% and 12% showing a lower level of personal accomplishment. Main reasons for this increase in emotional exhaustion in the Saudi physicians were: pressure and violence from the patients and attendants, disorganized patient flow, difficult paperwork and lack of cooperation between colleague doctors. Aggressive behavior by patients and their attendants, sometimes ending up in verbal and physical violence, were mostly noticed in the developing countries [13, 28]. Both doctors and nurses working in emergency departments or elsewhere are exposed to such conditions, compromising their safety and self-esteem [28]. Studies proved these hostile behaviors to be predictors of burnout, similar to our study findings where multivariate analysis showed patient pressure/violence to be the main predictor of overall burnout and emotional exhaustion. However, a study conducted in Qatar showed less patient pressure/violence, attributed to high literacy rate there [23]. Some other contributory factors may be the overburdened Saudi primary health care system, PHCCs managers' attitudes, and poor opportunities for career advancement.

The variation in burnout prevalence across different regions and countries can possibly be justified by the variation in culture, nature of health care system, and attitude of the patients and educational qualifications. Moreover, this can be due to differences in the assessment scales and study designs [27]. Demir, et al. in a study conducted in Turkey, identified age, years of experience, unsupervised work, and burden of work (especially number of patients per day) to be significant predictors of burnout [29]. Our study also revealed that patients in outpatient departments per day to be a significant predictor of burnout. Lagerström, et al. reported work dissatisfaction, doctor’s own health threats, and disequilibrium between family and work demands as some of the most important factors contributing to burnout among Iranian doctors and nurses [30]. However, as our study was conducted in PHCCs, which do not have night shifts, these factors were not reported.

Studies conducted in Cypriot [31] and South Africa [32] reported low salaries, difficulties in working in public sector hospitals with limited resources, difficult working schedules and understaffing as major predictors of burnout. A health survey covering 914 Australian and New Zealand junior doctors identified age and workload to be positive predictors of burnout [33]. Even though this present study was conducted in public sector setups, the participants were mostly satisfied with their salaries and working schedules. No significant difference in the overall burnout of FPs and GPs was noted in our study. However, Al-Shoraian, et al. [8] highlighted GPs having more burnout compared to FPs because of more work burden and additional duties. However, family medicine consultants were not satisfied with the limited facilities available at PHCCs that forced them to refer patients elsewhere. Similarly, no statistically significant correlation was noted between burnout with physician’s age, and between burnout with the number of working hours per day.

There are a few limitations in our study; first, participants were limited to mainly junior doctors and representation of senior doctors was less. Second, the internal validity of the study is limited by its cross-sectional design that was limited to doctors working in PHCCs only, making it difficult to understand the causal relationship.

## Conclusions

Emotional exhaustion is the most prominent feature of overall burnout in the physicians of the primary health care centers. The main reasons include patient pressure/violence, unorganized patient flow, fewer support services at the PHCCs, paperwork, and slow administrative transactions. Health education interventions during pre-employment training programs for prevention of burnout syndrome, and periodic screening system using aMBI questionnaire for early signs of burnout are recommended.

## References

[1] Battu N, Chakravarthy GK (2014). Quality of work life of nurses and paramedical staff in hospitals. Int J Bus Adm Res Rev.

[2] Ali M, Mohammad HY (2006). A study of relationship between managers’ leadership style and employees’ job satisfaction. Int J Health Care Qual Assur Inc Leadersh Health Serv.

[3] Allen D (2007). What do you do at work? Profession building and doing nursing. Int Nurs Rev.

[4] Garman AN, Evans R, Krause MK (2006). Professionalism. J Healthc Manag.

[5] Truzzi A, Wanderson S, Bucasioet E (2008). Burnout in a sample of Alzheimer’s disease caregivers in Brazil. Eur J Psychiatry.

[6] Ozyurt A, Hayran O, Sur H (2006). Predictors of burnout and job satisfaction among Turkish physicians. QJM.

[7] Shanafelt TD (2009). Enhancing meaning in work: a prescription for preventing physician burnout and promoting patient-centered care. JAMA.

[8] Al-Shoraian GMJ, Hussain N, Mohsen AF (2011). Burnout among family and general practitioners. Alexandria Journal of Medicine.

[9] Ashtari Z, Farhady Y, Khodaee MR (2009). Relationship between job burnout and work performance in a sample of Iranian mental health staff. Afr J Psychiatry (Johannesbg).

[10] Al-Sareai NS, Al-Khaldi YM, Mostafa OA (2013). Magnitude and risk factors for burnout among primary health care physicians in Asir Province, Saudi Arabia. East Mediterr Health J.

[11] Maslach Maslach, C C (1993). Burnout. A multidimensional perspective. Professional Burnout: Recent Developments in Theory and Research.

[12] Regehr C, Glancy D, Pitts A (2014). Interventions to reduce the consequences of stress in physicians: a review and meta-analysis. J Nerv Ment Dis.

[13] Lee RT, Seo B, Hladkyj S (2013). Correlates of physician burnout across regions and specialties: a meta-analysis. Hum Resour Health.

[14] Mata DA, Ramos MA, Bansal N (2015). Prevalence of depression and depressive symptoms among resident physicians: a systematic review and meta-analysis. JAMA.

[15] Abdo SA, El-Sallamy RM, El-Sherbiny AA (2015). Burnout among physicians and nursing staff working in the emergency hospital of Tanta University, Egypt. East Mediterr Health J.

[16] Maslach C, Schaufeli WB, Leiter MP (2001). Job burnout. Annu Rev Psychol.

[17] Zuraida AS, Zainal NZ (2015). Exploring burnout among Malaysian junior doctors using the abbreviated Maslach Burnout Inventory. Malaysian J Psych.

[18] Maslach C, Jackson SE, Leiter MP (1996). Maslach burnout inventory manual. 3rd ed. http://www.worldcat.org/title/maslach-burnout-inventory-manual/oclc/807246527?referer=di&ht=edition.

[19] Bakker AB, Killmer CH, Siegrist J (2000). Effort–reward imbalance and burnout among nurses. J Adv Nurs.

[20] Riley GJ (2004). Understanding the stresses and strains of being a doctor. Med J Aust.

[21] Deckard G, Meterko M, Field D (1994). Physician burnout: an examination of personal, professional, and organizational relationships. Med Care.

[22] Selaihem AA (2013). Prevalence of burnout amongst physicians working in primary care in Riyadh military hospital, Saudi Arabia. Int J Med Sci Public Health.

[23] Abdulla L, Al-Qahtani DM, Al-Kuwari MG (2011). Prevalence and determinants of burnout syndrome among primary healthcare physicians in Qatar. S Afr Fam Pract.

[24] Wu H, Liu L, Wang Y (2013). Factors associated with burnout among Chinese hospital doctors: A cross-sectional study. BMC Public Health.

[25] Asai M, Morita T, Akechi T (2007). Burnout and psychiatric morbidity among physicians engaged in end-of-life care for cancer patients: a cross-sectional nationwide survey in Japan. Psychooncology.

[26] Soler JK, Yaman H, Esteva M (2008). Burnout in European family doctors: the EGPRN study. Fam Pract.

[27] Kolstad HA, Hansen AM, Kargaard A (2011). Job strain and the risk of depression: is reporting biased?. Am J Epidemiol.

[28] Kalemoglu M, Keskin O (2006). Burnout syndrome at the emergency service. Scand J Trauma Resusc Emerg Med.

[29] Demir A, Ulusoy M, Ulusoy MF (2003). Investigation of factors influencing burnout levels in the professional and private lives of nurses. Int J Nurs Stud.

[30] Lagerström M, Josephson M, Arsalani N (2010). Striving for balance between family and work demands among Iranian nurses. Nurs Sci Q.

[31] Pavlakis A, Raftopoulos V, Theodorou M (2010). Burnout syndrome in Cypriot physiotherapists: a national survey. BMC Health Serv Res.

[32] Blaauw D, Ditlopo P, Maseko F (2017). Comparing the job satisfaction and intention to leave of different categories of health workers in Tanzania, Malawi, and South Africa. Glob Health Action.

[33] Markwell AL, Wainer Z (2009). The health and wellbeing of junior doctors: insights from a national survey. Med J Aust.

